# Pathophysiology of Primary Budd-Chiari Syndrome: A Narrative Review

**DOI:** 10.7759/cureus.96539

**Published:** 2025-11-11

**Authors:** Angie Katerine Montes López, Jhon Edwar Garcia Rueda, David Herrera Correa, Juan Carlos Restrepo Gutiérrez

**Affiliations:** 1 General Practice, University of Antioquia, Medellin, COL; 2 Internal Medicine, Pontifical Bolivarian University, Medellin, COL; 3 Faculty of Medicine, Pontifical Bolivarian University, Medellin, COL; 4 Hepatology, Hospital Pablo Tobón Uribe, Medellin, COL

**Keywords:** budd-chiari syndrome, hepatic venous outflow obstruction, hepatology, portal hypertension, thrombophilia mutations

## Abstract

Budd-Chiari syndrome (BCS) is a rare hepatic vascular disorder characterized by obstruction of hepatic venous outflow, leading to sinusoidal congestion, portal hypertension, and centrilobular necrosis - a classical model of postsinusoidal portal hypertension. This review provides an integrated analysis of the pathophysiology of BCS according to the site of obstruction (major hepatic veins, small/centrilobular veins, or inferior vena cava), describing how increased sinusoidal pressure, reduced portal flow, and compensatory mechanisms (intrahepatic collaterals, caudate lobe hypertrophy, and arterialization) shape clinical phenotypes that range from asymptomatic to acute, subacute, and chronic forms. The review highlights the etiopathogenesis of prothrombotic states - particularly myeloproliferative neoplasms (predominantly JAK2 V617F), hereditary thrombophilias, and acquired conditions, such as antiphospholipid syndrome and paroxysmal nocturnal hemoglobinuria - as well as the contributory role of pregnancy, the puerperium, and systemic diseases. It also discusses epidemiologic variability between Western and Asian populations and its clinical implications. Finally, it emphasizes a diagnostic approach based on Doppler ultrasonography (supported by computed tomography (CT) or magnetic resonance imaging (MRI)) and a stepwise management strategy prioritizing early anticoagulation, treatment of portal hypertension complications, decompression via transjugular intrahepatic portosystemic shunt (TIPS), and liver transplantation in selected cases. Understanding these mechanisms is key to improving diagnostic accuracy and guiding therapeutic decisions that have substantially enhanced patient survival.

## Introduction and background

Budd-Chiari syndrome (BCS) is a rare hepatic vascular disorder characterized by obstruction of hepatic venous outflow, which can occur at any level from the small hepatic veins to the junction of the inferior vena cava (IVC) with the right atrium [1,2]. This obstruction, in the absence of cardiac or pericardial causes, produces a hemodynamic disturbance that culminates in sinusoidal congestion, increased portal pressure, and centrilobular necrosis, constituting a classic model of postsinusoidal portal hypertension [3]. This hemodynamic alteration leads to hepatomegaly, ascites, and hepatic dysfunction due to congestion and ischemic injury. Over time, progressive fibrosis and cirrhosis may develop, highlighting BCS as a prototypical cause of secondary portal hypertension with significant morbidity and potential for liver failure.

BCS is classified as primary when the obstruction results from thrombotic occlusion of the hepatic veins or IVC, with stenosis or webs often representing sequelae of previous thrombosis, or secondary, when it derives from extrinsic compression or invasion by tumors, abscesses, or cysts [4,5]. The primary form is predominantly thrombotic and represents the hepatic manifestation of prothrombotic states, both hereditary (e.g., deficiencies of protein C, protein S, or antithrombin, or mutations in factor V Leiden and prothrombin) and acquired, with myeloproliferative syndromes being the most frequent causes [1,2]. In fact, up to half of patients present mutations in the JAK2 V617F gene, and in many cases, multiple thrombotic risk factors coexist [6].

The pathophysiology of the syndrome centers on the interruption of hepatic venous outflow, leading to increased sinusoidal pressure, decreased portal flow, and progressive hepatic congestion. Centrilobular hypoxia results in hepatocellular necrosis and stimulates activation of stellate cells, triggering perivenular fibrosis and, in chronic cases, congestive cirrhosis [3]. In acute phases, massive necrosis may cause fulminant hepatic failure, whereas in subacute or chronic forms, compensatory mechanisms such as the formation of intrahepatic collaterals and compensatory arterialization develop. These mechanisms mitigate congestion but promote the appearance of regenerative or dysplastic nodules, and even hepatocellular carcinoma [2].

Clinical manifestations depend on the degree and speed of obstruction, ranging from asymptomatic cases - reported in approximately 10-20% of patients - to acute hepatic failure with ascites and painful hepatomegaly [7]. This variability reflects the dynamic interaction between pathophysiologic mechanisms, the extent of vascular involvement, and the capacity to develop collateral pathways. Therefore, understanding the pathophysiology not only allows interpretation of clinical phenotypes but also guides therapeutic decisions - from anticoagulation to decompressive procedures such as TIPS or liver transplantation - within the stepwise approach proposed by international guidelines [7].

In recent decades, advances in molecular genetics, hemodynamic imaging, and interventional therapies have transformed the prognosis of this disease, with five-year survival rates approaching 90% in specialized centers [2]. However, questions remain regarding the mechanisms that determine chronicity, the uneven fibrosis between lobes, and the transition to hepatocellular carcinoma - features that make BCS a model for studying hepatic vascular pathophysiology.

In this context, the present narrative review aims to provide an updated and integrative overview of the pathophysiological mechanisms underlying primary BCS, emphasizing the hemodynamic consequences of hepatic venous outflow obstruction, the role of prothrombotic states such as myeloproliferative neoplasms and hereditary thrombophilias, and their correlation with clinical presentation and disease progression, as well as the diagnostic and therapeutic implications derived from these mechanisms.

## Review

Epidemiology

Global Overview

Budd-Chiari syndrome (BCS) is a rare condition with substantial geographic variation in its etiology and presentation. Its estimated prevalence is approximately 1 case per million population per year, with an annual incidence of 1 per million and an overall prevalence of 11 per million [6,8,9]. The mean age at diagnosis ranges from 35 to 40 years, with no significant sex predominance. In children, BCS accounts for 0.1% of liver diseases in Western countries, compared to up to 15% in Asian countries [7,8].

Western Countries

In Western populations, primary BCS - resulting from thrombosis of the hepatic veins - is the predominant form. The major hepatic veins are most often affected, and the underlying mechanism is usually thrombotic rather than membranous. Approximately 79-84% of patients have at least one prothrombotic disorder, and about one-third have multiple risk factors [10]. Among these, myeloproliferative neoplasms (MPNs) are the most frequent cause, occurring in 35-50% of Western patients. The Janus kinase 2 (JAK2) V617F mutation is present in up to half of these cases, while CALR and MPL mutations are less common [8,11].

Other relevant risk factors include use of oral contraceptives, antiphospholipid syndrome (APS), paroxysmal nocturnal hemoglobinuria, factor V Leiden, systemic inflammatory diseases such as sarcoidosis or connective tissue disorders, mastocytosis, multiple myeloma, and other hereditary thrombophilias (e.g., protein C and S deficiencies, factor II mutation, or antithrombin deficiency). Although local factors such as abdominal surgery, trauma, or intra-abdominal inflammatory conditions (e.g., biliary infection or pancreatitis) have been reported, their association with BCS is uncommon and likely coincidental rather than causative [6].

Eastern Countries

In contrast, studies from Asian countries, particularly China and India, have shown distinct patterns. The most frequent form is combined obstruction of the IVC and hepatic veins, often due to membranous occlusion or webs rather than pure thrombosis [8]. The prevalence of JAK2 V617F and other MPN-related mutations is markedly lower (2-4%) compared with Western cohorts [11-13]. Hereditary thrombophilias such as protein C, protein S, or antithrombin deficiencies also play a lesser role. Environmental and infectious factors, together with pregnancy-related or local inflammatory conditions, appear to contribute more prominently to disease development in these regions [11-13].

Prognosis

Without treatment, the three-year mortality of BCS is approximately 90%, mainly due to complications of portal hypertension and cirrhosis, such as variceal bleeding, refractory ascites, and hepatocellular carcinoma. With modern management - including anticoagulation, transjugular intrahepatic portosystemic shunt (TIPS), and liver transplantation when indicated - the five-year survival now exceeds 70% [14,15].

General pathophysiological mechanisms

The pathophysiology of BCS is based on hepatic venous obstruction, which may result, for instance, from thrombosis of the hepatic veins or the IVC to varying degrees. Residual thrombosis and fibrosis can lead to complete obliteration, focal segmental occlusion, or stenosis [8].

Subsequently, this obstruction causes increased sinusoidal pressure, portal hypertension, and ascites formation. A regional decrease in portal venous perfusion leads to hepatic congestion, hypoxia, ischemic necrosis, fibrosis development, and parenchymal atrophy. Abrupt occlusion may result in acute ischemia and severe hepatic dysfunction. If sinusoidal pressure is not relieved through therapeutic interventions or the development of collateral veins, regenerative nodules, fibrosis, and eventually cirrhosis develop [6,16].

Collateral veins develop within the liver as a compensatory mechanism for venous drainage. In addition to the right, middle, and left hepatic veins, accessory hepatic veins, such as the inferior right and caudate veins, also contribute to drainage. In cases of IVC obstruction, extrahepatic venous collateral pathways - including the azygos, hemiazygos, retroperitoneal, and thoracoabdominal wall veins - attempt to facilitate venous outflow. These changes occur gradually over months or years [8].

The asynchronous involvement of the hepatic veins and the resulting atrophy-hypertrophy mechanisms explain why a markedly dysmorphic liver is common. Hypertrophy of the central parts of the liver - mainly the caudate lobe - is partly related to the preservation of adequate venous drainage through the numerous small veins of the caudate lobe that drain directly into the IVC [2].

**Figure 1:**
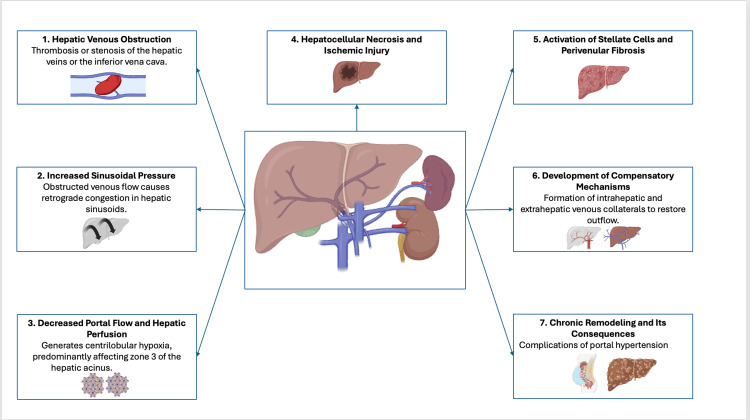
Pathophysiology of Budd-Chiari syndrome. Created by the authors (BioRender).

Hepatic fibrosis related to BCS predominantly occurs in the central portion of the hepatic lobule, with centro-central bridging and preservation of vascular architecture, unlike other forms of cirrhosis [16]. BCS is also recognized as a cause of hepatocellular carcinoma, which develops in approximately 5-15% of patients, particularly in those with long-standing disease and advanced hepatic fibrosis (Figure 1) [8].

Pathophysiology according to the type of obstruction

Primary BCS is classified into three types depending on the anatomical location of the venous obstruction [16], as follows:

Obstruction of the Major Hepatic Veins (Right, Middle, and Left Hepatic Veins)

Also referred to as the classic form of BCS, this is the most common presentation in Western countries and predominantly affects women. Thrombosis or stenosis of one or more major hepatic veins obstructs hepatic venous outflow into the IVC, leading to retrograde sinusoidal congestion, centrilobular necrosis, and, over time, progressive hepatic fibrosis.

From a clinical perspective, it manifests with painful hepatomegaly, ascites, and elevated transaminases, which may progress to hepatic failure if venous drainage is not restored. Persistent obstruction results initially in centrilobular fibrosis, followed by nodular regeneration, portal hypertension, and ultimately cirrhosis [4].

Small Hepatic Vein (Sinusoidal) Obstruction - Distinction From BCS

This rare variant involves the terminal or centrilobular hepatic venules, producing microvascular obstruction that causes sinusoidal stasis, centrilobular hypoxia, and hepatocellular necrosis. Although it may resemble BCS clinically, it is more accurately classified as sinusoidal obstruction syndrome (SOS) - formerly known as veno-occlusive disease (VOD) - rather than a true form of BCS. SOS is most often associated with chemotherapy-related hepatotoxicity (e.g., busulfan, cyclophosphamide) and presents with milder biochemical abnormalities and subacute hepatic dysfunction. For this reason, it is discussed here only for differential purposes and should not be confused with classical hepatic venous outflow obstruction [17].

Obstruction of the IVC

This form may occur with or without simultaneous involvement of the major hepatic veins. It is more common in men and in Asian regions, usually presenting as a chronic condition with milder symptoms [2]. The obstruction prevents hepatic venous drainage into the heart, leading to chronic hepatic congestion and the development of compensatory collateral circulation through the azygos, hemiazygos, and superficial abdominal veins. The clinical course is typically chronic and insidious, with milder manifestations such as mild ascites, discrete hepatomegaly, and dilation of superficial abdominal veins [4,18].

When both the IVC and the major hepatic veins are obstructed simultaneously, the condition is more severe, potentially presenting as acute fulminant hepatitis. In such cases, the hepatic parenchyma becomes markedly congested with fewer collateral drainage pathways, resulting in severe sinusoidal hypertension, decreased portal flow, and hepatocellular necrosis [4].

Etiopathogenesis and prothrombotic states

The pathogenesis of primary BCS is closely related to systemic or local prothrombotic conditions that predispose to hepatic venous thrombosis. These can be hereditary or acquired and are summarized below [16].

Primary BCS and Thrombosis

Primary BCS is regarded as the hepatic expression of underlying prothrombotic conditions [16]. Up to 88% of patients have identifiable acquired or hereditary causes; although in up to 20% of cases, no associated factor can be found - these are classified as idiopathic BCS [6-8].

The prothrombotic conditions most frequently associated with BCS, particularly the classic type, include the following: factor V Leiden mutation, prothrombin gene (PT) mutation, protein C and S deficiency, antithrombin deficiency, APS, hyperhomocysteinemia, and paroxysmal nocturnal hemoglobinuria (PNH) [16].

Myeloproliferative Neoplasms (MPNs)

MPNs are hematologic disorders arising from hematopoietic stem cells. They include polycythemia vera (PV), essential thrombocythemia (ET), and primary myelofibrosis (PMF) - collectively known as Philadelphia chromosome-negative MPNs [16].

Among all patients with BCS, 16-62% have an MPN, and PV is the most frequent subtype (18-43%), followed by ET (8-14%). In Europe, MPNs are the most common cause of classic BCS, accounting for 35-50% of cases (16), whereas in China, MPNs are reported in only 4-5% of patients [8].

The JAK2 V617F mutation is found in more than half of patients with MPNs. Calreticulin (CALR) mutations have been reported in 2.9% of BCS patients, and thrombopoietin receptor gene (MPL 515) mutations in 5% of patients with ET (8). It is mandatory to evaluate all patients with BCS for underlying mutations, even if blood counts are normal. A genetic MPN mutation panel - including JAK2 V617F, CALR, and MPL 515 - is recommended, as many patients may have masked polycythemia [8,16].

In 26% of patients, a diagnosis of MPN precedes BCS, while in 74%, BCS is the presenting manifestation of an undiagnosed MPN. Notably, 41% of BCS patients who were JAK2 V617F-positive but lacked hematologic features of MPN at diagnosis went on to develop overt MPN within seven years of the initial BCS diagnosis [8].

Hereditary Thrombophilias

Hereditary thrombophilias are mutations that increase the risk of thrombosis either due to defective neutralization of thrombin (e.g., antithrombin deficiency) or impaired control of thrombin generation (e.g., factor V Leiden, protein C and S deficiency, or prothrombin G20210A mutation) [16]. Factor V Leiden is the most common hereditary thrombophilia associated with BCS, occurring in 22-32% of Western patients and 17-26% of Indian patients [8].

Acquired Prothrombotic Conditions

Other acquired conditions, such as APS, hyperhomocysteinemia, and Behçet’s disease, may contribute to the development of BCS. APS appears to be the third most common prothrombotic factor in classic BCS in Western countries, with an estimated prevalence of antiphospholipid antibodies between 18% and 25% [5].

Among these antibodies, lupus anticoagulant is the most specific, followed by beta-2 glycoprotein I and anticardiolipin antibodies [8]. PNH is a rare acquired hematopoietic stem cell disorder, in which thrombosis is one of the major clinical manifestations - most frequently occurring in the splanchnic veins, particularly the hepatic veins (40.7%) [16].

Pregnancy and the Puerperium

Pregnancy and the puerperium have been reported more frequently in Asia than in Western countries. One study reported that most women diagnosed with BCS had at least two additional prothrombotic risk factors besides pregnancy, suggesting a possible interaction between pregnancy and pre-existing prothrombotic factors, resulting in an increased predisposition to develop BCS.

Many other systemic diseases are also associated with BCS, although less frequently. These include inflammatory bowel disease, sarcoidosis, systemic lupus erythematosus, mixed connective tissue disease, Sjögren’s syndrome, nephrotic syndrome, and protein-losing enteropathy [7,8].

Pathophysiologic-clinical correlation

From a pathophysiologic standpoint, three main mechanisms can be identified:

First: A phlebitic syndrome, manifested by abdominal pain, fever, malaise, and signs of systemic inflammation.

Second: Liver congestion predominantly affects the central region of the hepatic lobule. This leads to hepatic enlargement and massive, rapid formation of ascitic fluid. The rise in sinusoidal pressure also increases portal pressure, resulting in the development of collateral circulation between obstructed and adjacent regions.

Third: Decreased hepatic perfusion causes liver hypoxia, which is usually transient. This ischemia leads to centrilobular hepatocyte necrosis, as these cells are particularly susceptible to hypoxia. The hepatic injury in BCS results from both sinusoidal congestion and hypoxic ischemia; however, these processes are generally reversible and rarely fulminant, owing to several compensatory mechanisms. These include the development of collateral circulation by hepatocytes in response to increased sinusoidal pressure, compensatory hypertrophy of adjacent territories - particularly segment I (caudate lobe) - and elevated portal pressure, which enhances hepatic arterial flow. Clinical manifestations appear only when these compensatory mechanisms are overwhelmed. From a practical perspective, the disease can be clinically categorized into the following forms [8].

a. Asymptomatic Form

A phase characterized by the absence of ascites, hepatomegaly, and pain. At this stage, the compensatory mechanisms - especially the development of large collateral veins - have not yet been overcome by the degree of sinusoidal and portal hypertension, explaining the absence of clinical signs [17].

b. Symptomatic Forms

Acute Form: This form is rare and results from hepatic ischemia secondary to increased sinusoidal pressure. It is characterized by hepatic failure, sometimes severe but rarely fulminant or subfulminant, associated with ascites and often acute kidney injury, without significant hepatic morphological changes. Typical findings include painful hepatomegaly and transaminase elevations greater than five times the upper normal limit. This entity likely corresponds to simultaneous obstruction of all three hepatic veins in the absence of underlying liver disease. The prognosis depends on the course of the acute phase and, after six months, becomes similar to that of the chronic form [18].

Subacute Form:** **It is defined by a disease duration of less than two months with features of both acute hepatic congestion and early chronic remodeling. Typical findings include the rapid development of ascites, painful hepatomegaly, renal dysfunction, and mild-to-moderate jaundice. Transaminases are usually elevated more than five times the upper normal limit, and the INR is increased but generally remains above 40% of normal activity. Hepatic dysmorphia is often evident on imaging, reflecting the coexistence of acute ischemic injury and evolving fibrosis. This form tends to have a poorer prognosis than the chronic form [19].

Chronic Form:** **This form is characterized by typical hepatic dystrophy, a distinctive collateral venous network, transaminase levels below five times normal, and progressive development of ascites. Complications are mainly related to ascites, which may initially respond to diuretics or be refractory from the beginning. Portal hypertension-related complications such as esophageal varices occur in 5-15% of patients. Finally, hepatocellular carcinoma may develop as a rare complication due to progressive hepatic fibrosis and remodeling [8,9,19-22].

## Conclusions

BCS is a rare condition; however, it should be considered in any case of unexplained acute or chronic liver disease. It is often underdiagnosed, requiring a high index of suspicion in patients presenting with portal hypertension of unknown etiology. Screening for underlying mutations is mandatory, including a myeloproliferative neoplasm genetic panel assessing for JAK2V617F, CALR, and MPL 515 mutations, given their strong association with this group of disorders.

Obstruction of the major hepatic veins is the most common type of occlusion in the general population and may occur with or without concomitant obstruction of the inferior vena cava. Doppler ultrasound is the gold standard for diagnosis; however, it is operator dependent, so complementary imaging such as contrast-enhanced CT or MRI may be performed. Liver biopsy is not required for diagnosis. Anticoagulant therapy should be initiated as early as possible - unless contraindicated - and is sufficient in up to 18% of patients to halt disease progression. Complications such as portal hypertension should be managed according to clinical practice guidelines and recommendations established for cirrhosis. Hepatocellular carcinoma screening should be performed every six months using serum alpha-fetoprotein levels and liver ultrasound in patients without hepatic nodules or with multiple nodules identified on MRI.
